# Purinergic-mediated Ca^2+^ influx in *Dictyostelium discoideum*

**DOI:** 10.1016/j.ceca.2008.04.001

**Published:** 2008-12

**Authors:** Melanie J. Ludlow, David Traynor, Paul R. Fisher, Steven J. Ennion

**Affiliations:** a Department of Cell Physiology and Pharmacology, University of Leicester, PO Box 138, Leicester LE1 9HN, UK; b MRC Laboratory of Molecular Biology, Hills Road, Cambridge CB2 0QH, UK; c Department of Microbiology, La Trobe University, Melbourne, VIC 3086, Australia

**Keywords:** Purinergic, P2X, P2Y, *Dictyostelium*, ATP, ADP, Aequorin, Calcium

## Abstract

The presence of five P2X-like genes (*p2xA–E*) in *Dictyostelium* suggests that nucleotides other than cAMP may act as extracellular signalling molecules in this model eukaryote. However, p2xA was found to have an exclusively intracellular localisation making it unclear whether *Dictyostelium* utilise P2 receptors in a manner analogous to vertebrates. Using an apoaequorin expressing strain we show here that *Dictyostelium* do possess cell surface P2 receptors that facilitate Ca^2+^ influx in response to extracellular ATP and ADP (EC_50_ = 7.5 μM and 6.1 μM, respectively). Indicative of P2X receptor activation, responses were rapid reaching peak within 2.91 ± 0.04 s, required extracellular Ca^2+^, were inhibited by Gd^3+^, modified by extracellular pH and were not affected by deletion of either the single *Gβ* or *iplA* genes. Responses also remained unaffected by disruption of *p2xA* or *p2xE* showing that these genes are not involved. Cu^2+^ and Zn^2+^ inhibited purine-evoked Ca^2+^ influx with IC_50_ values of 0.9 and 6.3 μM, respectively. 300 μM Zn^2+^ completely abolished the initial large rapid rise in intracellular Ca^2+^ revealing the presence of an additional smaller, slower P2Y-like response. The existence of P2 receptors in *Dictyostelium* makes this organism a valuable model to explore fundamental aspects of purinergic signalling.

## Introduction

Sequencing of the *Dictyostelium* genome [1] has revealed a diverse complement of predicted cell surface receptors and ion channels, many with mammalian homologues, thereby increasing the interest in this amoeboid protozoan as a model eukaryotic organism. In addition to the seven well-characterised cAMP and cAMP-like receptors involved in chemotaxis during aggregation, 48 additional putative seven transmembrane receptors (7TM) are present [1,2] including receptors for glutamate [3] and GABA [4]. In terms of the complement of ion channels, an intriguing finding is the presence of a family of five *Dictyostelium* genes predicted to code for proteins with weak homology to vertebrate P2X receptors (Dictybase [5] genes: *p2xA*, *p2xB*, *p2xC*, *p2xD* and *p2xE*).

Vertebrate P2X receptors are cell surface cation selective ion channels gated by extracellular ATP and consist of seven distinct subtypes (P2X_1−7_), which assemble as functional homo- or hetero-trimeric channels. Vertebrate P2X receptors play a fundamental role in a wide array of physiological processes including neurotransmission, smooth muscle contraction, immune cell function, bone formation and platelet aggregation [6,7].

Recently *Dictyostelium p2xA* has been shown to produce a cell surface ATP-gated ion channel when exogenously expressed in the human embryonic kidney cell line HEK293 [8]. The native P2XA protein in *Dictyostelium* however was found to have an exclusively intracellular localisation being present on the contractile vacuole membrane where it plays a role in osmoregulation [8]. It is not clear therefore whether *Dictyostelium* utilises P2 receptors in the cytoplasmic membrane to sense extracellular nucleotides in a manner analogous to purinergic signalling in vertebrates.

In addition to ionotropic P2X receptors, vertebrate purinergic signalling is also mediated by metabotropic P2Y receptors. Unlike the P2X channels, P2Y receptors are 7TM G-protein coupled receptors and have a wider agonist profile responding to purines, pyrimidines and UDP–glucose [9]. Eight P2Y receptors have been characterised in mammals (P2Y_1,2,4,6,11–14_) [9–12] and these couple via G_q_/G_11_ to phospholipase C-β (P2Y_1,2,4,6_ and _11_), via G_i_/G_o_ to inhibit adenyl cyclase (P2Y_12,13_ and _14_) and to G_s_ to stimulate adenyl cyclase (P2Y_11_). Whilst the literature has tended not to classify the *Dictyostelium* cAMP receptors as P2 receptors, it could be argued that these correspond to *Dictyostelium* P2Y receptors since they are 7TM receptors activated by an extracellular purine ligand (cAMP). It is therefore possible that some of the orphan *Dictyostelium* 7TM receptors are activated by extracellular nucleotides other than cAMP.

Early studies performed prior to the discovery of P2 receptors demonstrated that extracellular ATP could enhance chemoattractant-mediated cell aggregation in *Dictyostelium* [13,14] and also increase basal uptake of ^45^Ca^2+^ [15]. However, these studies were performed on the basis of ATP being a substrate for extracellular kinases rather than a signalling molecule *per se* making interpretation of the results with respect to P2 receptor function unclear.

In light of these early studies and the more recent finding that the *Dictyostelium p2xA* gene codes for an intracellular protein [8] we sought to establish whether *Dictyostelium* possess cell surface P2 receptors for extracellular nucleotides. Vertebrate P2X receptors are permeable to Ca^2+^ [16] as are *Schistosoma mansoni* P2X [17] and *Dictyostelium p2xA* [8]. Activation of P2Y_1,2,4,6 and 11_ receptors also result in an increase in intracellular Ca^2+^ via activation of inositol 1,3,5-trisphosphate receptors leading to release of Ca^2+^ from intracellular stores. We therefore assessed P2 receptor function in *Dictyostelium* by utilising an apoaequorin expressing strain [18] to detect extracellular nucleotide-evoked increase in intracellular Ca^2+^ levels. Using this technique, we clearly demonstrate the presence of cell surface P2 receptors for extracellular ATP and ADP in *Dictyostelium*.

## Materials and methods

### Materials

Benzyl coelenterazine (h-CTZ) was from Lux Biotechnology. Adenosine 5′-triphosphate (ATP) magnesium salt, adenosine 5′-diphosphate (ADP), adenosine monophosphate (AMP), uridine 5′-triphosphate (UTP), adenosine, α,β-methyleneadenosine 5′-triphoshate (αβ-MeATP), 2′(3′)-*O*-(4-benzoylbenzoyl)adenosine 5′-triphosphate (BzATP), 2-(methylthio)adenosine 5′-triphosphate (2-MeSATP), suramin, pyridoxal phosphate-6-azo(benzene-2,4-disulfonic acid) (PPADS), apyrase and ATP bioluminescent assay kit were from Sigma. Pluronic F-127 was from Invitrogen. α-[^32^P]-dCTP was from Amersham.

### *Dictyostelium* transformation

Cells were harvested from axenic medium, washed twice in ice-cold H_50_ buffer (20 mM HEPES, 50 mM KCl, 10 mM NaCl, 1 mM MgSO_4_, 5 mM NaHCO_3_, 1 mM NaH_2_PO_4_·2H_2_O, pH 7.0), resuspended at 5 × 10^7^ cells/ml in H_50_ buffer and 105 μl electroporated (two pulses of 0.75 kV at 25 μF, BioRad Genepulser) with 15 μg of pPROF120 plasmid [18] in a 1 mm cuvette. Transformant selection at 30 μg/ml G418 commenced after 24 h.

### *p2xA* and *p2xE* gene disruption

The *p2xA*^−^ and *p2xE*^−^ strains were produced in Ax2 by homologous recombination using disruption vectors based on pLPBLP [19,20] (pDT23 and p2XEKO, respectively). Flanking homology regions were generated by polymerase chain reaction (PCR) with the following primer pairs: *p2xA* 5′-flanking region (1846 bp) forward primer: 5′-TAAAGGGCC CAACTTCATTGTCTCATTCTAGTCATAGTAATCCAATTG-3′; reverse primer: 5′-TGAAGTCGACCTAAACGTCTGTCTCTAATTCTTACAATCTTTACTGTAG-3′. *p2xA* 3′-flanking region (1678 bp) forward primer: 5′-ATGAACTAGTGAATAACAACTG ATGAAGGTGAAGATGTTTTATATAC-3′; reverse primer: 5′-GGGGCGGCCGCTACAAAATAAAATCAAAAATGACAATAACAACTATTTAAC-3′. *p2xE* 5′-flanking region (990 bp) forward primer: 5′-ACCTGGGCCCAAAGACTCATTATTTTCC-3′; reverse primer: 5′-ACCTAAGCTTTCTATTTTTGCAATTAATAATTAC-3′. *p2xE* 3′-flanking region (1526 bp) forward primer: 5′-AGTATGCGGCCGCTATGCCCCAAAGAAGTAG-3′ reverse primer: 5′-TCACCCCGCGGTCACCATTAACGAAACC-3′). Flanking homology regions were cloned on either side of the blasticidin S resistance cassette via the exogenous restriction enodnuclease sites introduced by the primers during amplification (*p2xA*: 5′ApaI–SalI and 3′ SpeI–NotI, *p2xE*: 5′ ApaI–HindIII and 3′ NotI–SacII). Disruption cassettes were liberated as ApaI/NotI and ApaI/SacII fragments, for *p2xA*^−^ and *p2xE*^−^, respectively, and 15 μg used to transform Ax2 amoebae. Two independent null strains were isolated from 96-well tissue culture plates in axenic medium [21], supplemented with vitamins (0.1 mg/l B_12_, 0.02 mg/l biotin and 0.2 mg/l riboflavin) plus 10 μg/ml blasticidin S as the selective agent. *p2xA* and *p2xE* gene disruptants among the many blasticidin resistant clones were identified by PCR, using oligonucleotides located outside the disruption cassette and within the blasticidin resistance cassette, with Southern blot analysis performed to verify a single site of integration.

### Southern blot analysis

NcoI or XbaI digested genomic DNA was probed with α-[^32^P]-dCTP labelled XhoI–EcoRV restricted BSR cassette. The membrane was pre-hybridised and hybridised in Church buffer (0.5 M NaHPO_4_, pH 7.2, 1 mM EDTA, pH 8.0, 7% SDS) at 65 °C then washed (5 × 20 min) with 0.2 × SSC (30 mM NaCl, 3 mM Na_3_C_6_H_5_O_7_·2H_2_O), 0.1% SDS at 65 °C. Hybridised bands were visualised by autoradiography.

### Cell culture

*Dictyostelium* Ax2 and DH1 and transformants derived from these strains were grown axenically in HL-5 media in shaken suspension (180 rpm) at 22 °C. Strains expressing apoaequorin and *iplA*/*p2xA*/*p2xE* null mutants were grown in media supplemented with 30 μg/ml G418 and 10 μg/ml blasticidin S, respectively. Minimal growth medium for DH1 was supplemented with 20 μg/ml uracil.

### In vivo aequorin reconstitution and measurement of purinergic responses

*In vivo* reconstitution of aequorin was achieved by incubating apoaequorin expressing strains in the presence of 5 μM h-CTZ, added from a 1000× suspension in methanol containing 20% (w/v) Pluronic 127. Cells (1 × 10^7^ per ml) were incubated for 6 h in HL-5 or for 7 h in MES–DB buffer (10 mM MES, 10 mM KCl, 0.25 mM CaCl_2_, pH 6.8) to permit measurement of responses in vegetative and aggregation competent cells, respectively. Cells were then washed twice and resuspended in MES–DB buffer at 2.2–3.3 × 10^6^ cells/ml prior to measurements. Desensitisation of P2 receptors during the preparatory steps necessitated allocating a recovery period of 1 h prior to measurement of ATP/ADP responses. Variations in the amount of desensitisation and rate of recovery prevented direct comparison of magnitude between experiments and strains by percentage aequorin consumption. Data were therefore normalised to a common response between experiments. Luminescence signals were recorded using a Lumat LB 9507 luminometer (Berthold Technologies), with 3 ml of cell suspension. Agonists (300 μl) were delivered using the auto-injector of the luminometer so as to standardise the speed and force of injection between experiments. Dependence on extracellular calcium was determined by suspending cells in nominally calcium-free MES–DB, or in MES–DB containing 50 μM CaCl_2_ with subsequent injection of ATP or buffer only control solutions containing increasing concentrations of CaCl_2_ (50 μM to 21.5 mM). PPADS, suramin and di/trivalent ions were added 10 min prior to measurement of ATP-induced responses. To overcome problems with solubility the inhibitory effect of copper ions was investigated using MES–DB buffer at pH 5.2.

### Ectonucleotidase activity

Phosphate released from the hydrolysis of 500 μM ATP or ADP was determined by optical absorbance of the phosphomolybdate complex at 700 nm (NOVOstar, BMG Labtech). Ax2 wild type cells (1 × 10^6^ cells/ml) or apyrase (0.01–1 U/ml) in MES–DB buffer were incubated at 22 °C for 20–60 min, the reaction was terminated with 2 V of 2% H_2_SO_4_, 0.5% SDS, 0.5% ammonium molybdate and the phosphomolydbate complex was reduced in 0.1% ascorbic acid.

### ATP release

To rule out the possibility that the force of agonist injection could cause cells to release ATP, aequorin assay conditions were replicated for Ax2 cells in the presence of luciferase–luciferin (ATP bioluminescent assay kit (Sigma)) according to the manufacturer's instructions.

### Data analysis

Peak areas and response kinetics were determined using WinWCP software (Dr. J. Dempster University of Strathclyde, Scotland). The area of the mean mechanosensitive “buffer” response for each experiment was subtracted from the area of agonist evoked responses prior to determination of EC_50_ values using GraphPad Prism software (San Diego, USA). Responses are expressed as means with standard errors and the indicated sample sizes.

## Results

### Extracellular ATP evokes an intracellular Ca^2+^ response in *Dictyostelium*

**Figure 1:**
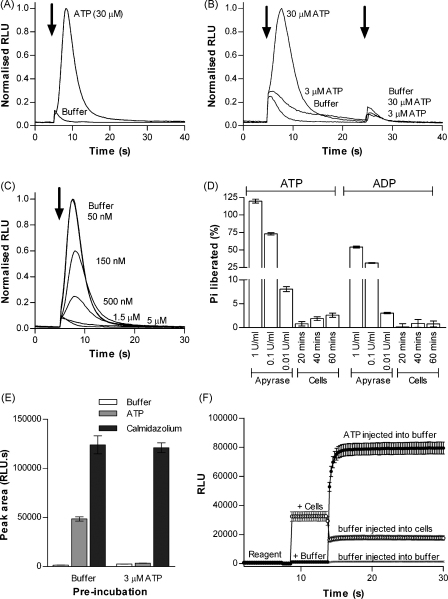
Measurement of ATP responses in apoaequorin expressing *Dictyostelium*. Apoaequorin expressing Ax2 (strain HPF275 [18]) cells were incubated with the cofactor benzyl coelenterazine in HL-5 media (vegetative cells; 1 × 10^7^ cells/ml) to reconstitute functional aequorin, transferred to MES–DB buffer (3.3 × 10^6^ (A and B) or 2.2 × 10^6^ (C and E) cells/ml) and responses to ATP or buffer were monitored using a luminometer. Traces are normalised to the 30 μM ATP response in each batch of cells. Arrows indicate time of agonist application (5 and 25 s). (A) Profile of luminescence in response to injection of 30 μM ATP and buffer (mechanosensitive response). (B) Desensitisation of response with two sequential injections of 30 μM ATP, 3 μM ATP and buffer (mechanosensitive response) at a 20 s interval. (C) Determination of ATP concentration required to desensitise responses. Following a 10 min preincubation with ATP at the indicated concentration or buffer (control), the response to 30 μM ATP was measured. (D) Assessment of ectonucleotidase activity by measurement of phosphate liberated from ATP/ADP hydrolysis by *Dictyostelium* (Ax2) (1 × 10^6^ cells/ml) or grade III apyrase (positive control, 20 min incubation). Values are expressed as phosphate liberated as a percentage of initial ATP/ADP concentration (500 μM) (mean ± S.E.M., *n* = 6). (E) Effect of 10 min preincubation with 3 μM ATP or buffer (control) on the magnitude of response to 30 μM ATP and 5 μM calmidazolium. Preincubation with ATP results in desensitisation of subsequent ATP responses but does not affect the calmidazolium response showing that aequorin depletion is not a factor in the desensitisation of ATP responses (mean ± S.E.M., *n* = 5). (F) In order to show that that the force of agonist injection does not cause ATP release from the cells, changes in extracellular ATP concentration were measured using a luciferase–luciferin ATP bioluminescent assay kit following injection of 150 μl buffer into 1.5 ml cells (3.3 × 10^6^ cells/ml in MES–DB), buffer injected into buffer (negative control) or 550 nM ATP injected into buffer (positive control). The mean of four independent experiments ± S.E.M. is shown.

In order to test the ability of extracellular nucleotides to evoke an intracellular calcium response we utilised the apoaequorin expressing Ax2 strain HPF275 [18]. After reconstitution of functional aequorin by incubation with the cofactor coelenterazine we observed a rapid and transient increase in intracellular Ca^2+^ upon exposure of both vegetative (Fig. 1A) and aggregation competent cells (Fig. 3A) to extracellular ATP. Similar to previous studies, we also observed a small rise in intracellular Ca^2+^ with the application of buffer alone (Fig. 1A). This buffer-evoked response has been attributed to the physical force of agonist application activating mechanosensitive ion channels [18,22]. As the onset of this mechanosensitive buffer response coincided with the onset of the nucleotide-evoked response, we compensated for the mechanosensitive component of ATP responses by obtaining a mean buffer response for each batch of cells and subtracting this from the area of the nucleotide-evoked responses (see Materials and methods). Responses to ATP were rapid and transient with a mean rise time of 2.91 ± 0.04 s from baseline to peak (*n* = 40, 100 μM ATP) and a mean decay of 6.13 ± 0.12 s (peak to 90% decay). Cells also remained refractory to subsequent applications of ATP (Fig. 1B). To further characterise this desensitisation we pre-incubated cells for 10 min with low concentrations of ATP prior to recording responses to 30 μM ATP in order to establish the concentration of ATP required to desensitise subsequent responses (Fig. 1C). Pre-incubation of cells with 1.5 μM ATP was sufficient to fully desensitise cells to a subsequent application of 30 μM ATP whereas pre-incubation with 50 nM ATP produced no detectable desensitisation with subsequent responses equivalent to those after control pre-incubation with buffer (Fig. 1C).

In order to rule out the possibility that desensitisation of ATP responses was due to aequorin depletion we pre-incubated cells in either buffer or 3 μM ATP and tested subsequent responses to either ATP or calmidazolium, a calmodulin antagonist known to evoke robust intracellular Ca^2+^ responses in *Dictyostelium* [23]. Pre-incubation of cells with 3 μM ATP abolished subsequent responses to 30 μM ATP but had no effect on subsequent calmidazolium responses demonstrating that depletion of aequorin was not the underlying cause of the desensitisation to repeated ATP applications (Fig. 1E). Prior to carrying out a pharmacological characterisation of nucleotide responses, it was necessary to determine whether *Dictyostelium* possess a significant ectonucleotidase activity since this could possibly distort results. To assess ectonucleotidase activity we utilised a molybdate-based ATPase assay to determine the amount of phosphate liberated when cells were incubated with ATP or ADP (500 μM) (Fig. 1D). After 20 min incubation with 1 × 10^6^ cells/ml only 0.8 ± 0.4% of ATP and 0.1 ± 0.6% of ADP was hydrolysed increasing to 2.6 ± 0.4% and 0.8 ± 0.6%, respectively after a 1 h incubation (Fig. 1D) showing that ectonucleotidase activity was unlikely to affect subsequent pharmacological characterisation of ATP responses. Since physical force such as shear stress on endothelial cells can stimulate cells to release ATP [24], another potential confounding factor in our experimental system was the possibility that the force of agonist injection could result in ATP release. This possibility was ruled out by monitoring ATP release using firefly luciferase. Injection of buffer into a cell suspension produced no detectable increase in luciferase activity whereas injection of 50 nM ATP was readily detected (Fig. 1F).

### ADP is equipotent to ATP and acts at the same receptor

**Figure 2:**
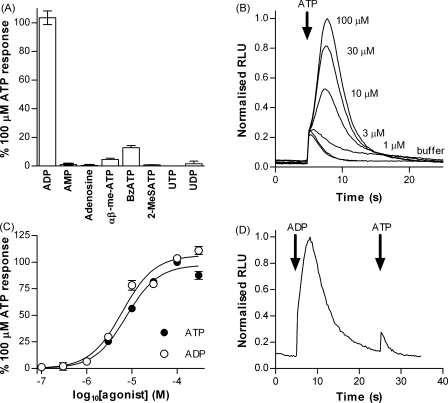
ADP and ATP are equipotent in eliciting an intracellular Ca^2+^ response. Responses to extracellular nucleotides were measured in vegetative aequorin-expressing Ax2 cells (3.3 × 10^6^ cells/ml). Arrows indicate time of injection (5 and 25 s). (A) Response to 100 μM ADP, AMP, adenosine, αβ-me-ATP, BzATP, 2-MeSATP, UTP and UDP, relative to 100 μM ATP (mean ± S.E.M., *n* = 5). (B) Representative traces showing concentration dependent response to ATP. (C) Concentration response curves for ATP and ADP. Data are normalised to the response to 100 μM ATP (mean ± S.E.M., *n* ≥ 5 from at least two independent experiments). (D) Cross desensitisation between ADP and ATP responses. 100 μM ATP was applied 20 s after 100 μM ADP.

Having established that extracellular ATP evoked a rise in intracellular Ca^2+^ in *Dictyostelium* and determined the suitability of the aequorin system for analysing nucleotide-evoked responses, we next tested a range of potential nucleotide agonists (Fig. 2A). ADP produced responses virtually identical to those evoked by ATP and weak responses were observed with the ATP analogues αβ-me-ATP (4.6 ± 1.8% ATP response) and 2′,3′-*O*-(4-benzoylbenzoyl)-ATP (BzATP) (12.7 ± 3.5% ATP response). No significant response was observed with AMP, adenosine, UTP, UDP or 2-MeSATP (all tested at 100 μM). The response to both ATP and ADP was concentration dependent with pEC_50_ values of 5.13 ± 0.05 (7.5 μM) and 5.21 ± 0.05 (6.1 μM), respectively (Fig. 2B and C). The Hill slopes for both the ATP and ADP concentration response curves were also very similar being 1.23 ± 0.13 and 1.21 ± 0.13, respectively. In order to investigate whether ADP and ATP were acting at the same receptor, we tested the ability of ADP to cross-desensitise the response to ATP. When ATP was applied immediately after an ADP evoked response, no ATP response above the level of the mechanosensitive buffer response was observed suggesting that ADP and ATP do indeed act through the same receptor (Fig. 2D).

### Responses to extracellular ATP and ADP require extracellular Ca^2+^ and are inhibited by gadolinium ions

**Figure 3:**
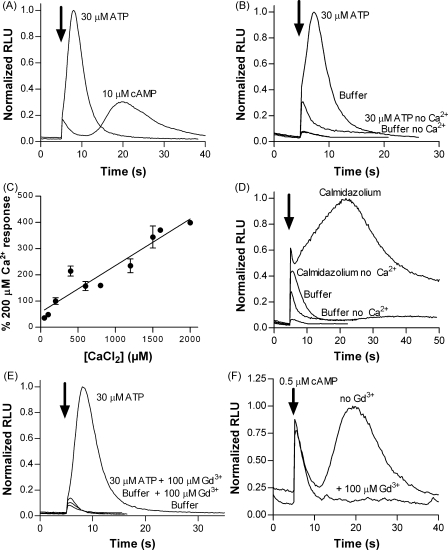
The purinergic response has faster kinetics than responses to cAMP, requires extracellular Ca^2+^ and is inhibited by Gd^3+^. (A) Cells were made aggregation competent by incubation in MES–DB buffer for 7 h and a direct comparison of responses to 30 μM ATP and 10 μM cAMP made. Traces are normalised to the 30 μM ATP response. Arrow indicates time of agonist injection (5 s). (B) Decreased magnitude of ATP responses in the absence of extracellular Ca^2+^ in vegetative cells. Responses to 30 μM ATP and buffer (mechanosensitive response) are shown for vegetative cells suspended in MES–DB buffer containing 0 μM (nominally calcium-free) or 250 μM Ca^2+^. (C) Magnitude of responses to 30 μM ATP with varying concentrations of extracellular CaCl_2_ in vegetative cells. Data are normalised to the response in 200 μM CaCl_2_ (mean ± S.E.M., *n* = 4 from three independent experiments). (D) Response of vegetative cells to 5 μM calmidazolium in MES–DB buffer containing 0 μM (nominally calcium-free) or 250 μM, CaCl_2_. Note that the initial rapid response to calmidazolium remains in the absence of extracellular Ca^2+^ confirming that aequorin is still capable of detecting rises in intracellular Ca^2+^ when extracellular Ca^2+^ is removed. (E) Inhibition of the response to 30 μM ATP in vegetative cells by gadolinium ions (100 μM). (F) Inhibition of the response to 0.5 μM cAMP in aggregation competent cells by 100 μM gadolinium ions. Traces are normalised to the maximum peak value. Arrows indicate time of injection (5 s).

Extracellular nucleotides can activate two distinct families of receptor, ionotropic P2X ligand gated ion channels and metabotropic P2Y seven transmembrane receptors [25]. We next sought to determine whether the *Dictyostelium* intracellular Ca^2+^ response to extracellular ATP and ADP is mediated by an ionotropic (P2X) or metabotropic (P2Y) mechanism. As an initial indicator we directly compared the time course of the response to ATP to that of cAMP, an agonist known to act via a family of four seven transmembrane receptors [26]. ATP responses had a faster onset than those of cAMP and decayed back to baseline more rapidly (Fig. 3A) suggesting that the mechanism leading to increased intracellular Ca^2+^ differs between these two agonists. The response to ATP required the presence of extracellular Ca^2+^ since responses were severely attenuated when experiments were carried out in nominally free Ca^2+^ buffer (Fig. 3B) and the amplitude of the response increased with increasing extracellular Ca^2+^ concentrations (Fig. 3C). Attenuation of the ATP response was not due to the absence of extracellular Ca^2+^ affecting the function of aequorin since control applications of calmidazolium in the absence of extracellular Ca^2+^ still showed the initial rapid peak component of the response (Fig. 3D). The presence of gadolinium, a trivalent ion known to block both P2X [27] and calcium channels, abolished the response to ATP (Fig. 3E) and in agreement with previous reports [18,28], also abolished responses to cAMP (Fig. 3F).

### The *iplA* and *Gβ* genes are not required for the purinergic response

**Figure 4:**
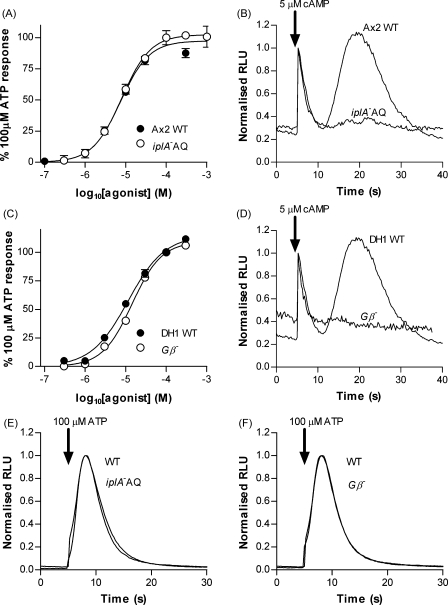
The purinergic response does not require the *iplA* or *Gβ* genes. (A) Concentration response curves for ATP in vegetative Ax2 and *iplA*^−^AQ strains. Data are normalised to 100 μM ATP for each strain (mean ± S.E.M., *n* ≥ 5 from two independent experiments). (B) Response to 5 μM cAMP in Ax2 and *iplA*^−^AQ aggregation competent cells. Similar to the parent strain *iplA*^−^, *iplA*^−^AQ does not exhibit a Ca^2+^ response to cAMP. Traces are normalised to the peak of the mechanosensitive response (first peak) in each strain. (C) Concentration response curves for ATP in vegetative DH1 and *Gβ*^−^ strains. Data are normalised to 100 μM ATP for each strain (mean ± S.E.M., *n* ≥ 5 from two independent experiments). (D) Loss of response to 5 μM cAMP in *Gβ*^−^AQ cells. Traces are normalised to the peak of the mechanosensitive response for each strain. (E and F) Representative responses to 100 μM ATP in vegetative *iplA*^−^AQ (E) and *Gβ*^−^AQ (F) cells compared to wild type. The kinetics of the ATP response is unaffected by deletion of either gene. Traces are normalised to the peak response for each strain.

Disruption of the *Dictyostelium* inositol 1,3,5-trisphosphate receptor-like gene *iplA* abolishes the rise in intracellular Ca^2+^ in response to cAMP [29]. We therefore tested whether disruption of *iplA* had any effect on the response to extracellular ATP or ADP. The *iplA*^−^ strain was transformed with the aequorin plasmid pPROF120 [18] to produce the strain *iplA*^−^AQ. Similar to the parent *iplA*^−^ strain, intracellular Ca^2+^ responses to cAMP were abolished in *iplA*^−^AQ (Fig. 4B). The response to ATP however was unaffected with an overlapping concentration response curve to that of wild type and a pEC_50_ of 5.10 ± 0.05 (7.9 μM) (Fig. 4A). The time course of the ATP response in the *iplA*^−^AQ strain was also identical to wild type (Fig. 4E). Having ruled out a role of the *iplA* gene in mediating the purinergic response we next asked whether the *Gβ* gene was required as indication of the involvement of G-protein coupled receptors. Intracellular Ca^2+^ responses to both folate and cAMP have been shown to be abolished in the *Gβ* null strain HPF330 [22] and we confirmed this for cAMP in our experimental set-up (Fig. 4D). Since the parent strain for HPF330 is DH1, it was first necessary to establish whether an ATP response was also present in this strain since our previous studies had utilised the Ax2 strain. ATP was slightly less potent in the DH1 strain compared to Ax2 (pEC_50_ = 4.94 ± 0.04 (11.4 μM)). However there was no significant difference between ATP concentration response curves for wild type DH1 and the mutant *Gβ*^−^ strain (*Gβ*^−^ pEC_50_ = 4.84 ± 0.03 (14.3 μM)) (Fig. 4C). Similarly, the time course of the ATP response was also unaffected by deletion of the *Gβ* gene (Fig. 4F) demonstrating that the mechanism of response does not involve a seven transmembrane receptor linking to *Gβ*.

### The *p2xA* and *p2xE* genes are not required for the purinergic response

**Figure 5:**
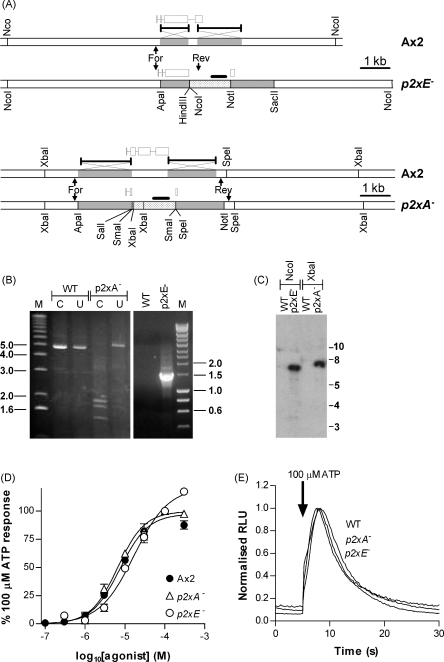
Disruption of the *p2xA* and *p2xE* genes by homologous recombination. (A) Homologous recombination strategy for disruption of the *p2xA* and *p2xE* genes. Generation of the *p2xA*^−^ strain left 190 bp of 5′ and 56 bp of 3′ coding sequence flanking the blasticidin S transferase cassette (bsr) (dotted bar). For *p2xE*^−^ 314 bp between intron 3 and exon 4 were replaced by bsr. (B) PCR screening of genomic DNA for blasticidin S resistant transformants; primer binding sites indicated by arrows in A. For *p2xA*^−^ an increase in size from 4947 to 5260 bp of the uncut (U) PCR amplicon and the presence of SmaI restriction sites (C), located within bsr, to produce bands of 1974, 1812 and 1474 bp are indicative of targeted insertion. For *p2xE*^−^ the introduction of a reverse primer binding site within bsr is indicative of targeted insertion. (D) NcoI (*p2xE*^−^) and XbaI (*p2xA*^−^) digested genomic DNA (10 μg) was hybridised with α-[^32^P]-dCTP labelled XhoI–EcoRV cut bsr probe (solid bars in A). Single bands of the expected size (7595 and 7028 bp) for *p2xA*^−^ and *p2xE*^−^ respectively, with no hybridisation to WT DNA, confirm a single site of insertion. (D) Concentration response curves for ATP for WT (Ax2), *p2xA*^−^ and *p2xE*^−^ strains. Data are normalised to 100 μM ATP for each strain (mean ± S.E.M., *n* ≥ 5 from two independent experiments). (E) Representative responses to ATP (100 μM) for *p2xA*^−^ and *p2xE*^−^ strains. The kinetics of the response to ATP is unaffected by deletion of either gene.

The rapid time course of the purinergic response, a requirement for extracellular Ca^2+^, inhibition by Gd^3+^ ions and no requirement for the *Gβ* or *iplA* genes are all indicative of an ionotropic (P2X)-mediated mechanism of Ca^2+^ influx rather than a metabotropic (P2Y)-mediated mechanism. *Dictyostelium* possesses a family of five genes predicted to encode proteins with homology to vertebrate P2X ion channels and one member of this family (*p2xA*) has recently been shown to be localised to intracellular membranes on the contractile vacuole system where it plays a role in osmoregulation [8]. Furthermore, when this gene was expressed on the extracellular membranes of HEK293 cells it formed an ATP-gated ion channel [8]. We investigated the possible role of the *p2xA* gene in mediating the *Dictyostelium* response to extracellular nucleotides along with that of another member of the *Dictyostelium* P2X-like gene family, *p2xE*. Mutant strains were generated using homologous recombination to disrupt either the *p2xA* or *p2xE* genes (Fig. 5A). The response to extracellular ATP remained in both these mutant strains with pEC_50_ values of 5.18 ± 0.05 (6.5 μM) for *p2xA*^*−*^ cells and 4.77 ± 0.06 (16.9 μM) for *p2xE*^−^ cells (Fig. 5D). The time course of the ATP response for these mutant strains was also very similar to wild type (Fig. 5E).

### Pharmacological properties of the purinergic response

**Figure 6:**
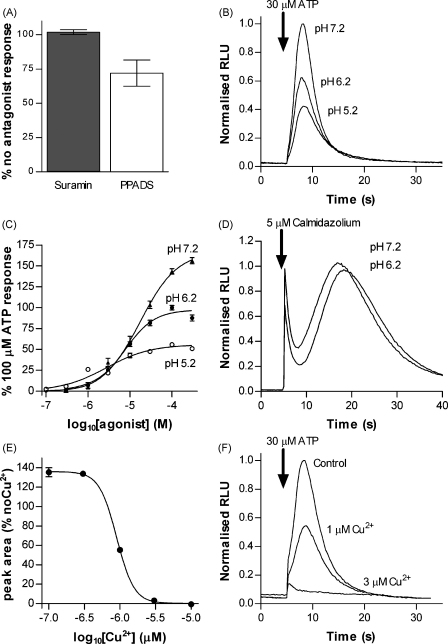
Pharmacological properties (A) response to 30 μM ATP in the presence of the P2 receptor antagonists suramin (100 μM) and PPADS (10 μM). Data are presented as mean ± S.E.M. (*n* = 5). Suramin does not antagonise the response to ATP. Note, the reduction in response in the presence of PPADS is likely due to absorbance of aequorin luminescence by PPADS (see Results). (B) Representative traces demonstrating the effects of extracellular pH on responses to 30 μM ATP. (C) Concentration response curves for ATP-induced responses recorded in extracellular buffer of pH 5.2, 6.2 and 7.2, data are normalised to 100 μM ATP at pH 6.2 (mean ± S.E.M., *n* ≥ 5 from at least two experiments). (D) Response to 5 μM calmidazolium is unchanged at pH 7.2 and 6.2 showing that the function of aequorin was unaffected. (E) Concentration dependent reduction in 30 μM ATP response by CuCl_2_ (mean ± S.E.M., *n* = 5 from two separate experiments). (F) Representative traces showing inhibition of the ATP response by copper. Traces are normalised to the control peak value.

Vertebrate P2 receptors are broadly blocked by suramin and pyridoxal-phoshate-6-azophenyl-2′,4′-disulphonate (PPADS) [7]. Suramin however had no effect on the purinergic response in *Dictyostelium* (tested at 100 μM). Responses in the presence of 10 μM PPADS were reduced by ∼25% (Fig. 6A). However, we measured the absorbance spectrum for this compound and found that it strongly absorbed light between 400 and 550 nm. Since this corresponds to the emission spectra for aequorin, it is likely that the reduction in measured response was due to absorbance of light emitted rather than specific inhibition.

Extracellular pH is known to modulate the function of several vertebrate P2X channels [30,31]. Increasing extracellular pH from 6.2 to 7.2 increased the maximum of the *Dictyostelium* ATP response by 66.7 ± 6.0% and decreased the pEC_50_ from 5.13 ± 0.05 (7.5 μM) at pH 6.2 to 5.56 ± 0.08 (2.7 μM) at pH 7.2. Conversely, decreasing pH to 5.2 reduced the maximum response by 44.1 ± 2.2% and increased the pEC_50_ to 4.75 ± 0.05 (17.6 μM) (Fig. 6B and C). This modulation by pH was not due to a non-specific effect of pH affecting the cells or the function of aequorin since control applications of calmidazolium remained unaffected by the same pH change (Fig. 6D).

Since micromolar concentrations of copper ions inhibit some mammalian P2X receptors and have been shown to block function of *Dictyostelium p2xA* when expressed in HEK293 cells [8], we tested the effect of this divalent ion on the endogenous *Dictyostelium* response to extracellular ATP. Copper inhibited the response with a pIC_50_ value of 6.05 ± 0.02 (0.9 μM) (Fig. 6E and F).

### Inhibition by Zn^2+^ reveals a slower component of the ATP response

**Figure 7:**
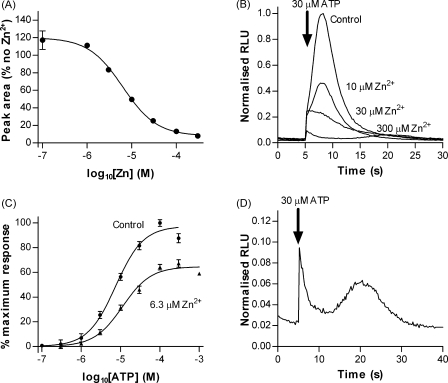
Inhibition by zinc reveals a smaller slower component to the ATP response. (A) Concentration response curve showing inhibition by extracellular Zn^2+^ of the response to 30 μM ATP (pIC_50_ 5.20 ± 0.06 (6.27 μM)) (mean ± S.E.M., *n* = 5 from two separate experiments). (B) Concentration response curves for ATP in the presence of an IC_50_ concentration of Zn^2+^ (6.3 μM) (mean ± S.E.M., *n* ≥ 5). (C) Representative traces showing inhibition of the response to 30 μM ATP by extracellular Zn^2+^. (D) The residual response to 30 μM ATP in the presence of 300 μM Zn^2+^ reveals a slower component of the response normally masked by the larger rapid response (expanded from C). Arrows indicate time of injection (5 s).

The divalent ion Zn^2+^ also blocked the *Dictyostelium* response to ATP with a pIC_50_ value of 5.20 ± 0.06 (6.3 μM) (Fig. 7A). The concentration response curve for ATP in the presence of an IC_50_ concentration of Zn^2+^ displayed a pEC_50_ similar to that in the absence of Zn^2+^ (4.95 ± 0.05 (11.2 μM) but with a reduced maximal response (Fig. 7C). 300 μM Zn^2+^ completely abolished the initial rapid and transient rise in intracellular Ca^2+^ (Fig. 7B). Removal of this larger rapid component of the response by Zn^2+^ inhibition revealed the presence of a smaller, later and more prolonged response that reached a peak 15 s after agonist application (Fig. 7D).

## Discussion

The discovery of a family of five P2X-like genes in *Dictyostelium* raised the possibility that nucleotides other than cAMP may act as extracellular signalling molecules in this model eukaryotic organism. In this study we have utilised an apoaequorin expressing strain to determine whether extracellular nucleotides can elicit purinergic signalling. We demonstrate that *Dictyostelium* do indeed possess cell surface purinergic receptors that facilitate a rapid increase in intracellular Ca^2+^ in response to extracellular ATP and ADP. This rapid purine evoked increase in intracellular Ca^2+^ likely represents a different phenomenon to the ATP-induced increase in basal ^45^Ca^2+^ uptake described in 1980 [15] since in this early study increases in ^45^Ca^2+^ were observed 5 and 10 min after ATP application with the rate of ^45^Ca^2+^ uptake declining to below baseline at 15 and 20 min post-stimulation. In our study, we describe a far more rapid calcium influx that begins immediately after ATP addition, peaks within 3 s and returns to baseline within 20 s after agonist application. This rapid purinergic response required extracellular Ca^2+^ (Fig. 3B and C) and was blocked by gadolinium ions (Fig. 3E). Whilst indicative of a P2X-mediated response, these two observations in themselves do not necessarily rule out P2Y receptor activation since cAMP acting via 7TM receptors also produces a response dependent on extracellular Ca^2+^ which can be blocked by gadolinium ions [18]. The time course of the purinergic response however was far more rapid than the response to cAMP (Fig. 3A) suggesting that a different mechanism is involved. Indeed the onset of the purinergic response coincided with the onset of the rapid “buffer” response which has been attributed to the direct activation of mechanosensitive ion channels [18,22], suggesting the purinergic response involves a direct Ca^2+^ influx rather than an indirect P2Y-mediated activation of a Ca^2+^ channel. Furthermore, whilst ablation of the *iplA*, or *Gβ* genes abolished responses to cAMP, the purinergic response is unaffected (Fig. 4) demonstrating that neither IP_3_ receptor mediated release of Ca^2+^ from internal stores nor activation of a 7TM receptor linking to *Gβ* is involved.

The endogenous *Dictyostelium* purinergic response to extracellular nucleotides shows some pharmacological properties similar to the previously described *Dictyostelium* intracellular P2X receptor *p2xA* [8] including insensitivity to antagonism by suramin and PPADS, which is unusual for P2 receptor mediated responses, inhibition by Cu^2+^ ions and weak activation by the ATP analogue BzATP. However, there was a marked difference in agonist sensitivity with ATP being a more potent activator of the endogenous Ca^2+^ response (EC_50_ ∼ 7.5 μM) compared to P2XA (EC_50_ > 200 μM) [8]. Furthermore ADP was equipotent to ATP at the endogenous response (ADP EC_50_ ∼ 6.1 μM), whereas it is not an agonist of P2XA [8]. This difference in agonist sensitivity and the fact that P2XA displays an exclusively intracellular localisation [8] make it unlikely that P2XA contributes to the response to extracellular nucleotides. We confirmed this by showing that ablation of the *p2xA* gene by homologous recombination had no effect on the endogenous purinergic response. Similarly we also mutated the *Dictyostelium p2xE* gene and again the purinergic response remained. Three other P2X-like genes are present in the *Dictyostelium* genome (*p2xB*, *p2xC* and *p2xD*) and it is therefore possible that one or a combination of these are involved in mediating the response to extracellular purines.

Whilst the dominant response to extracellular ATP/ADP showed characteristics indicative of P2X receptor activation, there was also a very small component of the response with a much slower onset. Normally this small slower component was masked by decay of the large P2X-like response. However when the P2X-like component was blocked with 300 μM Zn^2+^ a slower component became apparent (Fig. 7D). Apart from the four cAMP receptors, *RpKA* which is thought to bind conditioned media factor [2] and *GrlE* that binds GABA [4], ligands for the *Dictyostelium* 7TM receptor family are largely unknown. It is therefore possible that in addition to P2X receptors, *Dictyostelium* also possess 7TM P2Y receptors activated by extracellular ATP. The time course of the slower purinergic component was similar to that observed for cAMP which is known to act via 7TM receptors. However, the slow purinergic response was also observed in the *Gβ*^−^ strain in the presence of 300 μM Zn^2+^ (data not shown) raising the intriguing possibility that direct, G-protein independent, activation of a Ca^2+^ channel by a P2Y-like receptor could be involved.

The physiological function of purinergic signalling to extracellular ATP and ADP in *Dictyostelium* is not known. Using the under-agar chemotactic assay we were unable to detect any chemotactic response to ATP or ADP (data not shown). However, whilst not directly chemotactic in itself ATP has been shown to reduce the time to onset of cell aggregation, in response to starvation, in several species of *Dictyostelium* [13,14]. A possible function, therefore, could be in the modulation of chemotaxis to cAMP. However, unlike the cAMP response, which is only apparent in aggregation competent starved cells, an ATP evoked increase in intracellular Ca^2+^ calcium was observed in both aggregation competent and axenically growing cells, which are unresponsive to cAMP. Also, in contrast to the ADP response observed in this study, the enhancement of aggregation by ATP is not mimicked by ADP [13,14].

In this study we demonstrate that, in addition to the previously described intracellular P2XA receptor, *Dictyostelium* also possess cell surface purinergic receptors for extracellular nucleotides, which upon activation facilitate a rapid increase in intracellular Ca^2+^ concentration. *Dictyostelium* therefore represent a valuable model system to explore fundamental aspects of purinergic signalling in a eukaryotic organism with a relatively simple and well-annotated genome that is readily amenable to genetic manipulation and has the potential for powerful functional screens.

## Conflict of interest

None.
